# Antiasthmatic Effects of Herbal Complex MA and Its Fermented Product MA128

**DOI:** 10.1155/2012/769508

**Published:** 2011-11-15

**Authors:** Dong-Seon Kim, Seung-Hyung Kim, Bok-Kyu Kim, Min Cheol Yang, Jin Yeul Ma

**Affiliations:** ^1^Herbal Medicine Improvement Research Center, Korea Institute of Oriental Medicine, Daejeon 305-811, Republic of Korea; ^2^Institute of Traditional Medicine & Bioscience, Daejeon University, Daejeon 300-716, Republic of Korea

## Abstract

This study was conducted to determine if oral administration of the novel herbal medicine, MA, and its *Lactobacillus acidophilus* fermented product, MA128, have therapeutic properties for the treatment of asthma. Asthma was induced in BALB/c mice by systemic sensitization to ovalbumin (OVA) followed by intratracheal, intraperitoneal, and aerosol allergen challenges. MA and MA128 were orally administered 6 times a week for 4 weeks. At 1 day after the last ovalbumin exposure, airway hyperresponsiveness was assessed and samples of bronchoalveolar lavage fluid, lung cells, and serum were collected for further analysis. We investigated the effect of MA and MA128 on airway hyperresponsiveness, pulmonary eosinophilic infiltration, various immune cell phenotypes, Th2 cytokine production, OVA-specific IgE production, and Th1/Th2 cytokine production in this mouse model of asthma. In BALB/c mice, we found that MA and MA128 treatment suppressed eosinophil infiltration into airways and blood, allergic airway inflammation and AHR by suppressing the production of IL-5, IL-13, IL-17, Eotaxin, and OVA-specific IgE, by upregulating the production of OVA-specific Th1 cytokine (IFN-**γ**), and by downregulating OVA-specific Th2 cytokine (IL-4) in the culture supernatant of spleen cells. The effectiveness of MA was increased by fermentation with *Lactobacillus acidophilus*.

## 1. Introduction

Asthma is a chronic, complex respiratory disease caused by various airway obstructions, airway eosinophilic inflammation, and bronchial hyperresponsiveness [1]. It is a global health problem that results from a complex interplay between genetic and environmental factors [2] and excess production of Th2 cytokines (IL-4, IL-5, IL-13) relative to the Th1 cytokine IFN-*γ*. Eosinophils have a crucial role in the pathogenesis of allergic diseases. Clinical and experimental studies have established eosinophilia as a marked sign of allergic disorders [3]. IL-4 can directly induce airway hyperresponsiveness and airway and blood eosinophilia in asthmatic patients [4], and other investigators have shown an inhibitory effect of IFN-*γ* on pulmonary allergic responses [5]. CD4^+^ T cells play a crucial role in immune protection through their capacity to help B cells make antibodies, to recruit neutrophils, eosinophils, and basophils to sites of inflammation, and, through their production of cytokines and chemokines, to orchestrate immune responses [6]. Suppression of cytokine production in activated CD4^+^ T cells may be useful for the treatment of asthma. Th2 cytokines produced by CD4^+^ T cells, such as interleukin-4 (IL-4), IL-5, and IL-13, enhance immunoglobulin E (IgE) production and eosinophil accumulation, and IL-13 directly enhances mucus hypersecretion and AHR [7, 8]. Therefore, suppression of Th2 cytokine production in activated CD4^+^ Th cells may be useful for the treatment of inflammatory immune diseases including asthma.

To develop a novel herbal medicine for treatment of allergy, MA was prepared with herbs that were traditionally used to treat diseases related to antiallergy and anti-inflammatory. Recent studies have suggested that fermentation of herbal extract may have therapeutic advantages due to the increased absorption and bioavailability of the active components in the body [9–11]. To increase the antiallergic efficacy of MA, we fermented it with *Lactobacillus acidophilus*, which is naturally found in human and animal GI tract, mouth, and vagina and is most commonly used as a probiotic; we then evaluated antiasthmatic properties of MA and its fermented product, MA128, on airway eosinophil accumulation, Th2 cytokine production, various immune cell phenotypes, and histology in a murine model of asthma.

## 2. Materials and Methods

### 2.1. Animals

Five-week-old female BALB/c mice were obtained from Orient Bio Co. Ltd. (Seongnam, Republic of Korea). The experimental protocols used in the study have been approved by the committee for animal welfare at Daejeon University. Moreover, all animal procedures were conducted in accordance with the guidelines of the Institutional Animal Care and Use Committee of the South Korea Research Institute of Bioscience and Biotechnology (Daejeon, Republic of Korea).

### 2.2. Preparation of MA and MA128

All herbal plant materials were purchased from the Korea Medicine Herbs Association (Yeongcheon, Korea). All voucher specimens were deposited in the herbal bank of Herbal Medicine Improvement Research Center, Korea Institute of Oriental Medicine. A mixture of medicinal herbs (1840 g) consisting of *Sophora flavescens Aition*, *Glycyrrhizae Radix*, *Arctii Fructus*, *Cnidii Rhizoma*, and* Polygoni Cuspidati Radix*, and so forth, was boiled in 18.40 L of distilled water for 3 h using a Herb Extractor (Kyungseo, Korea), and the extract was filtered using standard testing sieves (150 *μ*m) to yield 15.7 L of the decoction. A 5 L portion of the decoction was freeze-dried to give the dried extract, MA (131.4 g), which was stored in desiccators at 4°C until use. For fermentation of MA, a 10 L portion of the decoction was adjusted to pH 8.0 using 1 M NaOH and then sterilized in an autoclave at 121°C for 5 min. Pure cultures of *Lactobacillus acidophilus *(KFRI 128) were obtained from the Korea Food Research Institute (KFRI). Before experimental use, the bacterial strain was incubated in MRS medium (10 g/L Peptone, 10 g/L Beef extract, 5 g/L Yeast extract, 20 g/L Glucose, 1 mL/L Tween 80, 2 g/L K_2_HPO_4_, 5 g/L Sodium acetate, 2 g/L Triammonium citrate, 0.2 g/L MgSO_4_·7H_2_O, 0.2 g/L MnSO_4_·4H_2_O, pH 6.2–6.6, Becton Dickinson and Company, Hunt Valley, Md, USA) at 37°C for 24 h. The MA (10 L) was inoculated with 10 mL of *Lactobacillus acidophilus *(1 × 10^8^ CFU/mL), and fermented at 37°C for 48 h. The fermented MA was then filtered through a 60-*μ*m nylon net filter (Millipore, Billerica, Mass, USA) and freeze-dried to give the dried fermented extract, MA128 (261.8 g), which was stored in desiccators at 4°C until use. 

### 2.3. HPLC Analysis of MA and MA128

The analytical performance liquid chromatography (HPLC) data were obtained on an Elite Lachrom analytical HPLC PDA system that included an L-2130 HPLC pump, an L-2200 autosampler, column oven (L-2350), and diode array UV/VIS detector (L-2455). The output signal of the detector was recorded using EZchrom Elite software for Hitachi. For sample separation, an OptimaPak C18 column (5 *μ*m, 100 Å, 4.6 mm × 250 mm, RS tech, Republic of Korea) was used. The mobile phase was water and acetonitrile containing 1% glacial acetic acid in a gradient elution at a flow rate of 1.0 mL/min and the column temperature was maintained at 40°C (Table 1). The injection volume of each sample was 10 *μ*L. To prepare analytical samples, MA and MA128 powders were accurately weighed and dissolved in 100% H_2_O at a concentration of 40 mg/mL. Prior to analysis, the sample was filtered through a 0.45-*μ*m filter.

### 2.4. Ovalbumin (OVA) Sensitization and Inhalation

As the modified protocol previously described [12], OVA (500 *μ*g/mL) in PBS was mixed with equal volumes of 10% (w/v) aluminum potassium sulfate (alum; Sigma) in distilled water, then incubated for 60 min at RT after adjustment to pH 6.5 using 10 N NaOH, and centrifuged at 750 ×g for 5 minutes. The OVA/alum pellet was resuspended to the original volume in distilled water. All mice were immunized on three different days (first day of 2, 3, 4 weeks before inhalational exposure) by intraperitoneal (I.P.) injections of 0.2 mL alum-precipitated antigen containing 100 *μ*g of OVA (Sigma-Aldrich Korea, Republic of Korea) bound to 4 mg of aluminum hydroxide (Sigma-Aldrich Korea, Republic of Korea) in PBS. Seven days after the second sensitization by intratracheal injection of 250 *μ*g of OVA (on day 21) on the back of the tongue, mice were exposed to aerosolized OVA for 30 min/day, 3 days/week for 5 weeks (at a flow rate of 250 L/min, 1% OVA in normal saline for first 4 weeks, and 2% OVA in normal saline for last 1 week). MA (300 mg/kg/day), MA128 (400, 300, 200 mg/kg/day), and cyclosporin A (CsA, 10 mg/kg/day) in solution form were orally administered 3 times a week for the last 5 weeks. One day after the last OVA exposure (2% OVA inhalation), airway hyperresponsiveness was determined and samples (bronchoalveolar lavage fluid, lung cells, and serum) were collected for further molecular analyses.

### 2.5. Bronchoalveolar Lavage Fluid (BALF)

Immediately following assessment of AHR, mice were sacrificed by I.P. injection of sodium pentobarbitone (100 mg kg^−1^). The trachea was cannulated and BAL was obtained by washing the airway lumina. Briefly, cells in the lungs were recovered by flushing 1 mL of BAL fluid (1 mM EDTA, 10% FBS, PBS) into the lungs via the trachea. Total cell numbers were counted, and 100 *μ*L of fluid was cytospun onto glass slides using a Cytospin centrifuge (Cellspin, Hanil, Republic of Korea; 400 g for 4 minutes). Differential cell counts were performed after staining with a Diff-Quik Stain Set (Baxter Healthcare Corp., Miami, Fla, USA). The supernatant of BALF was stored at −25°C for determination of cytokine levels.

### 2.6. Digestion of Pulmonary Tissue and Cell Preparations

Single cell suspensions from lung tissues and BALF were isolated by mechanical disruption in RPMI 1640 medium supplemented with 2 mM L-glutamine, 100 U/mL penicillin, 100 *μ*g/mL streptomycin, 50 *μ*M 2-mercaptoethanol, 20 mM HEPES, and 2% heat-inactivated fetal bovine serum (FBS, GIBCO, Grand Island, NY, USA). Briefly, the lungs were removed from thoracic cavity. After mincing using sterile scalpels, the tissue was incubated in PBS containing 1 mg/mL Collagenase IV and 2 mg/mL Dispase for 40 min at 37°C in a sterile polypropylene tube. After incubation, lung tissue was vigorously pipetted up and down to further dissolve remaining tissue clumps and then filtered using a 70 *μ*m cell strainer (Falcon, Le Pont de Claix, France). The total number of cells was counted manually using a hemocytometer chamber (Fisher). Between 2 and 4 × 10^3^ cells were spun onto glass slides (Cytospin centrifuge, Cellspin, Hanil, Republic of Korea; 400 g for 4 minutes). Differential counts were assessed according to standard morphologic criteria.

### 2.7. Determination of Airway Hyperresponsiveness (AHR)

Airway hyperresponsiveness in mice was estimated using a previously described method with modifications [13]. A Buxco system (Biosystem XA; Buxco Electronics Inc, Troy, Conn, USA) was used to evaluate the extent of airway constriction in different groups of mice following the protocol described previously. Penh is equal to Pause × PEF/PIF, where Pause = (Te − *Tr*⁡)/Tr (PIF, peak inspiratory flow; PEF: peak expiratory flow; Te, expiratory time; Tr, relaxation time). In this experiment, mice were aerosolized with OVA for 30 min/day, 3 days/week for 5 weeks. At 24 hours after the final inhalation, mice were given aerosolized normal saline, followed by 3.15, 6.25, 12.5, 25, and 50 mg/mL methacholine (Sigma) serially. Airway reactivity was then monitored for 30 minutes. Differences in Penh value between groups were evaluated using an unpaired Student's *t*-test.

### 2.8. Hematoxylin-Eosin (H&E), Masson-Trichrome (M-T), and Periodic Acid-Schiff (PAS) Staining

BALB/c mice were injected, inhaled, and sprayed with OVA for 5 weeks (three times a week) to induce asthma. Three experimental groups were treated with MA (300 mg/kg), MA128 (300 mg/kg), or CsA (10 mg/kg) for the last 4 weeks (daily). At the end of the experiment, the lungs were removed and analyzed histologically using a modified protocol previously described [14]. Briefly, the lung tissue was embedded in paraffin then cut into 3 *μ*m thickness sections for staining with H&E solution or MT solution. The tissue was subsequently mounted and coverslipped with Dako-mounting medium (Dakocytomation; Carpinteria, Calif, USA). The degree of airway inflammatory cell infiltration was scored in a double-blind screen by two independent researchers. The degree of peribronchiole and perivascular inflammation was evaluated on a subjective scale of 0–2 as previously described [15]. Periodic acid-Schiff (PAS) staining was performed to identify mucus secretion in lung tissue. Frozen sections (30 mm in thickness) of each tissue were prepared. Each sample section was mounted on the gelatin-coated slide, stained with PAS reagents, dehydrated, and coverslipped with the permount. The PAS-positive goblet cells were counted manually and normalized to the length of the bronchial epithelial perimeter on the basal side, and expressed as the number of PAS-positive cells per mm of basement membrane.

### 2.9. Antibodies and Flow Cytometric Analysis

All antibodies (such as anti-CD3, CD4, CD8, CCR3, CD69, B220, CD23, CD11b, and Gr-1) for flow cytometric analysis were purchased from Becton Dickinson (BD) PharMingen (San Diego, Calif, USA). Cells from lung tissues and BALF were stained with the indicated antibodies in staining buffer (PBS containing 1% FBS and 0.01% NaN3) for 10 min on ice and analyzed by two-color flow cytometry on an FACSCalibur using CellQuest software (BD Biosciences, Mountain View, Calif, USA).

### 2.10. Enzyme-Linked Immunosorbent Assay (ELISA)

Interleukin (IFN- *γ*, IL-4, IL-5, IL-13, IL-17, eotaxin, etc.) production in BALF and anti-OVA IgE in serum of the indicated mice (*n* = 8) was measured by ELISA according to the manufacturer's instructions with a monoclonal antibody-based mouse interleukin ELISA kit (R&D system). OVA-specific IL-4 and IFN-*γ* were produced by spleen cells suspended in RPMI 1640 medium supplemented with 2 mM L-glutamine and 5% fetal bovine serum. The spleen cells were cultured for 48 hrs at a concentration of 1 × 10^5^ cells/well in 96-well culture plates (Corning Inc., Cambridge, Mass, USA) with or without 10 *μ*g/mL of OVA in a humidified atmosphere of 5% CO_2_ in air at 37°C. The culture supernatants were collected and assayed for IFN-*γ* and IL-4 antibodies induced by OVA using ELISA. All data represent the mean and standard deviation from at least three separate determinants and were compared by analysis of variance (ANOVA).

### 2.11. Statistical Analysis

Data were analyzed by one-way analysis of variance (ANOVA) or unpaired Student's *t*-test followed by Dunnett's multiple comparison test (SPSS version 14.0 statistic software). The difference between the normal group and the control group (OVA + vehicle) was clearly distinguished, but statistical significance between the normal group and the control group was not shown in the figures and tables to emphasize the statistical differences between the experimental groups and the control group. Results (presented as mean ± standard error of mean) were considered statistically significant if *P *values were <0.05^*∗*^, <0.01^*∗∗*^, or <0.001^*∗∗∗*^.

## 3. Results

### 3.1. HPLC Chemical Fingerprinting of MA and MA128

**Figure 1:**
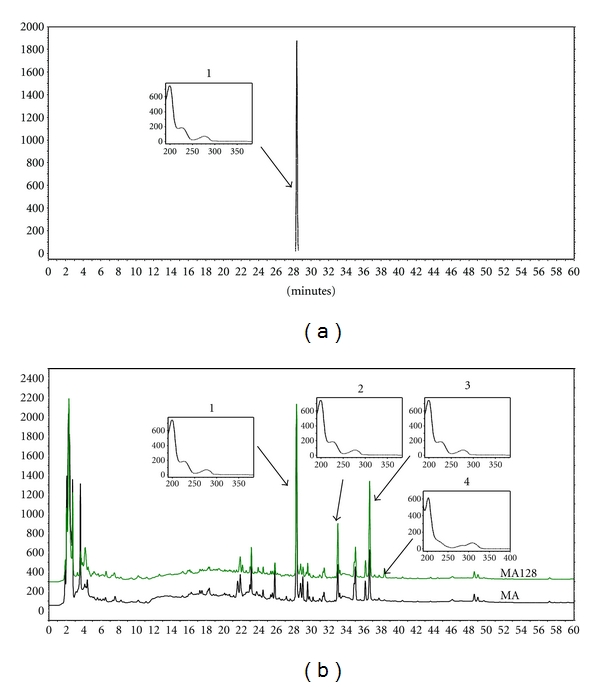
The HPLC fingerprinting of MA and MA128 at 203 nm. (a) Arctiin standard; (b) MA and MA128. 1. Arctiin, *t*_*R*_ 28.41 min; 2. not identified, *t*_*R*_ 33.12 min; 3. not identified, *t*_*R*_ 36.8 min; 4. not identified, *t*_*R*_ 38.5 min.

In this study, an HPLC method was developed to achieve chemical fingerprinting of MA and MA128. Arctiin was used as a marker standard as it was the highest peak in the HPLC chromatograms of MA and MA128; it was isolated using column chromatography and identified using NMR and MS in preliminary study. A binary gradient system consisting of water-acetonitrile as mobile phase was able to separate the compounds in the extracts and the best wavelength for the detection was 203 nm as it showed the most peaks including the marker and main peaks (Figure 1). Arctiin (peak 1) appeared at 28.41 min and three other major peaks (peak 2-3) appeared at 33.12, 36.8, and 38.5 min, respectively.

### 3.2. Inhibitory Effect of MA and MA128 on Airway Hyperresponsiveness (AHR)

**Figure 2:**
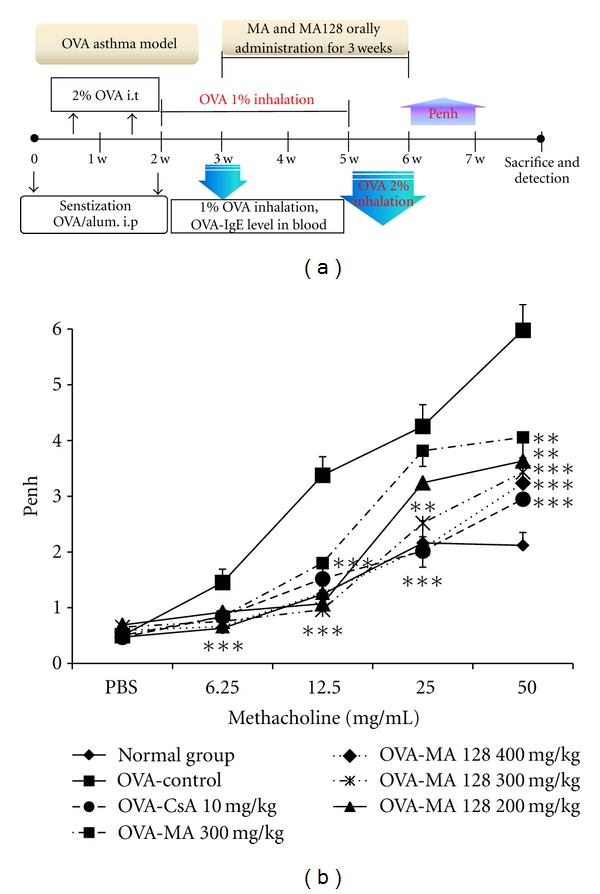
Effect of MA and MA128 on airway hyperresponsiveness. (a) Schematic diagram of methacholine-induced AHR in the sensitization protocol; (b) PenH was measured with a Buxco box, as described in Materials and Methods. Results are expressed as mean ± S.E. (*N* = 8). Statistical significance between control and treatment groups was assessed by ANOVA (*P < 0.05, **P < 0.01, ***P < 0.001). Normal group: Normal Balb/c mice, OVA-Control: Ovalbumin inhalation + vehicle, OVA + CsA (10 mg/kg), OVA + MA (300 mg/kg), OVA + MA128 (400 mg/kg, 300 mg/kg, 200 mg/kg).

In order to evaluate the inhibitory effect of CsA, MA, and MA128 on airway hyperresponsiveness, total pulmonary airflow in mice was estimated using whole-body plethysmography. Penh was measured using a Buxco system on day 1 after final inhalation, and samples were immediately collected. Methacholine treatment is useful to demonstrate the distinct effect of drugs on Penh value by way of inducing AHR. In OVA-sensitized/-challenged mice, the dose-response curve of Penh value was shifted to the left compared with that of normal mice (Figure 2(b)). As shown in Figure 2(b), relative to animals sensitized with OVA (control group), AHR to methacholine was significantly reduced in the MA-treated (300 mg/kg) group (*P* < 0.05), MA128-treated (400, 300 and 200 mg/kg) groups (*P* < 0.001, *P* < 0.01, and *P* < 0.05, resp.), and CsA-treated (10 mg/kg) group (*P* < 0.001). All of the MA128 (400, 300, and 200 mg/kg) groups exhibited dose-dependent inhibitory effects that were stronger than that of the MA-treated (300 mg/kg) group.

### 3.3. Histological Analysis of Lung Sections

**Figure 3:**
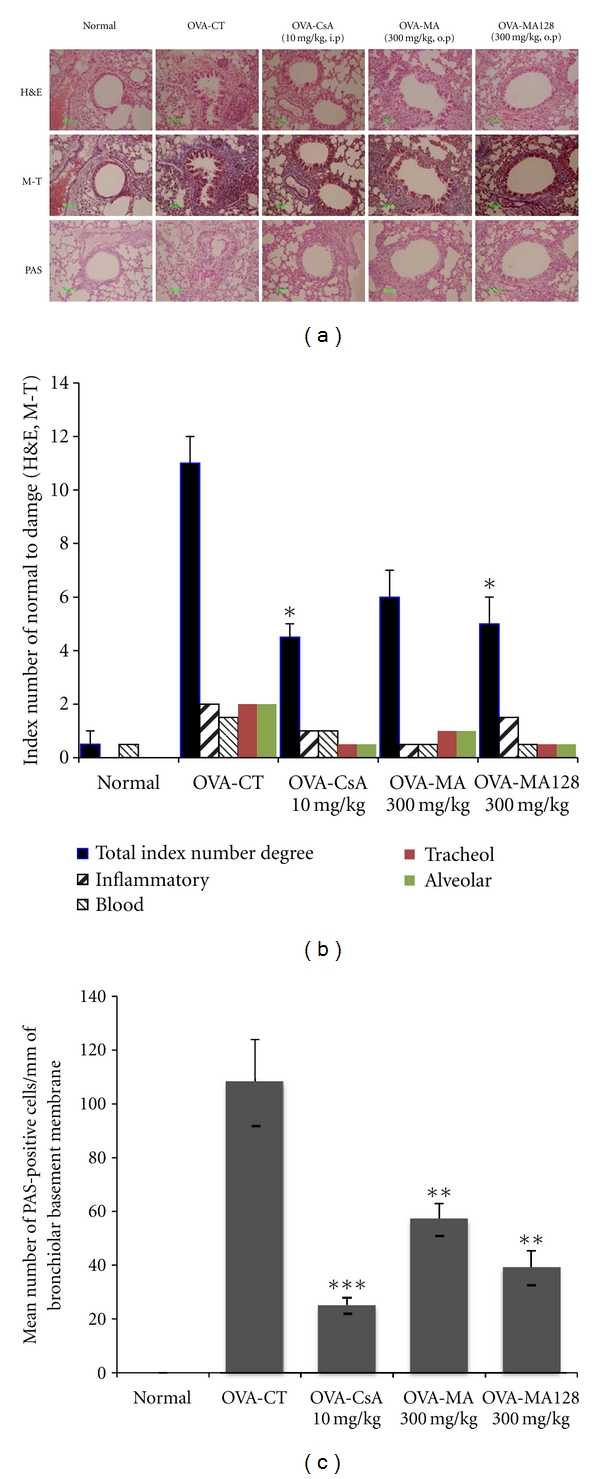
Histological analysis of lung sections. (a) Effect of MA and MA128 on histology of lung tissue (H&E, M-T and PAS staining) in lung cells of the OVA-induced murine model of asthma; (b) total index number of normal to damage (0 to 2); (c) mean number of PAS-positive cells/mm of bronchiolar basement (PAS staining). H&E: hematoxylin-eosin stain, M-T: Masson trichrome stain, PAS: periodic acid-Schiff stain, Normal: normal Balb/c mice, OVA-CT: Ovalbumin inhalation + vehicle, OVA + CsA (10 mg/kg), OVA + MA (300 mg/kg), OVA + MA128 (300 mg/kg). *P < 0.05, **P < 0.01, ***P < 0.001 for control group versus treatment groups.

The histopathological investigation of OVA-challenged mice and MA, MA128, and CsA-treated mice revealed inflammatory changes compared with saline-challenged normal mice. Also, we observed infiltration of leukocytes in histologic sections of lungs from OVA-challenged control mice, and lung tissue sections from OVA-challenged mice showed a distinct inflammatory infiltrate and erosion in peribronchial and perivascular areas. The peribronchial and perivascular inflammatory infiltrate consisted of eosinophils and mast cells, admixed with lymphocytes. Eosinophil infiltration was mainly observed in the peribronchial regions of the lung. In contrast, histological sections from MA-treated (300 mg/kg) and MA128-treated (300 mg/kg) mice and CsA-treated (10 mg/kg) mice revealed reduced airway inflammation in lung tissue (Figures 3(a) and 3(b)). The degrees of goblet cell hyperplasia and mucus hyperproduction were evaluated by means of PAS staining and quantification of PAS-stained cells. The OVA-challenged control mice showed significantly increased mean numbers of PAS-positive cells when compared with saline-challenged normal mice. In particular, there were greater reductions in the mean number of PAS-stained goblet cells in the MA and MA128-treated and CsA-treated asthmatic mice than in OVA-sensitized/-challenged mice (Figures 3(a) and 3(c)). Therefore, MA128 treatment showed better lung anti-inflammatory efficacy than MA treatment.

### 3.4. Inhibitory Effect of MA and MA128 on Airway Eosinophil Accumulation and Influx of Inflammatory Cells into Lung and BALF

**Figure 4:**
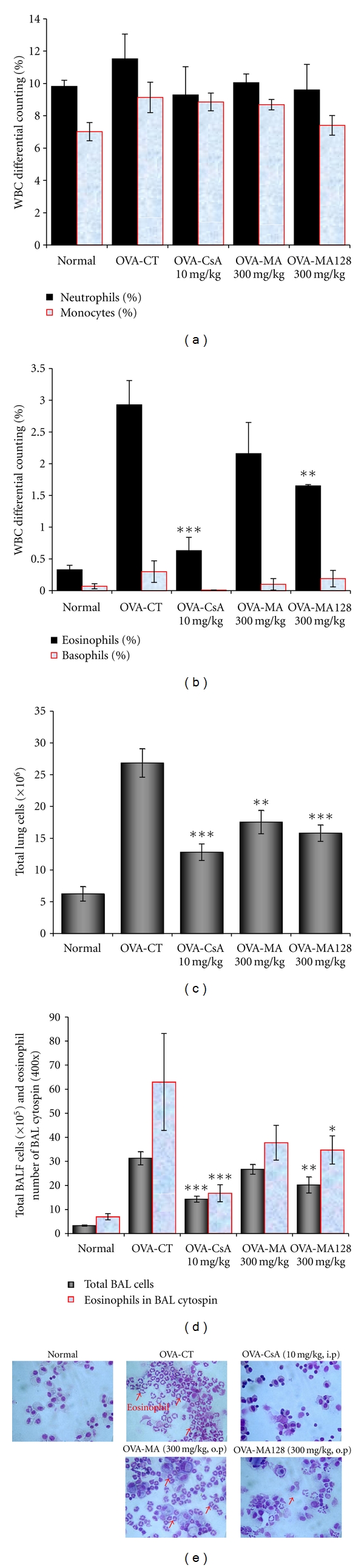
Effect of MA and MA128 on airway inflammatory cells. (a), (b), and (c) Effects of MA and MA128 on total neutrophils, monocytes, eosinophils, and basophils in blood and total lung cells, total leucocytes and eosinophils in BAL. As described in Section 2, whole blood was harvested 24 hrs after the last OVA challenge; (d) total inflammatory cell numbers in blood were counted, and a minimum of 200 cells were classified as eosinophils or lymphocytes; (e) photomicrograph (original magnification, ×200) of BALF cytospins from a sensitized mouse exposed repeatedly to allergen (Diff-Quik Stain). Results are expressed as mean ± SE (*N* = 8). Statistical significance between control and treatment groups was assessed by ANOVA (*P < 0.05, **P < 0.01, ***P < 0.001). Normal: normal Balb/c mice, OVA-CT: Ovalbumin inhalation + vehicle, OVA + CsA (10 mg/kg), OVA + MA (300 mg/kg), OVA + MA128 (300 mg/kg).

The number of total leukocytes in the BALF obtained from the PBS saline-challenged group was 3.3 ± 0.25 × 10^4^ cells, indicating that few eosinophils were detected in this group. On the other hand, the total number of leukocytes (31.3 ± 2.75 × 10^4^) and eosinophils in the BALF cytospin of the OVA-challenged was 10 times higher than that in the PBS saline-challenged group. The total number of leukocytes was significantly reduced in MA and MA128-treated (300 mg/kg) and CsA-treated mice compared with control mice, and the number of total lung cells was also significantly reduced in MA and MA128-treated (300 mg/kg) and CsA-treated mice (Figure 4(c)). MA128 (300 mg/kg) and CsA (10 mg/kg) also decreased the total number of eosinophils in BALF (Figure 4(d), and accompanied by changes of eosinophils counts in BALF (Figure 4(e)).

### 3.5. Inhibitory Effect of MA and MA128 on Absolute Number of Immune Cell Subtypes in Murine OVA-Induced Asthma Lung and BALF

To evaluate the effect of MA and MA128 on T and B cell subtypes, flow cytometric analysis was performed. The numbers of CD4, CD8, CCR3, CD69, B220, CD23, CD11b, Gr-1 positive cells in the lungs of OVA-challenged mice were increased compared to those in the saline-treated group, and generally, each value from MA and MA128-treated (300 mg/ kg) and CsA-treated mice were significantly lower than those from OVA-challenged mice (Table 2). Similarly, CsA administration resulted in significant reduction in T cell subtypes. The effects of MA and MA128 (300 mg/kg) and CsA treatment on leukocyte subsets in lungs and BALF included marked changes in the numbers of CD4^+^ helper T cells, CD8^+^ c/s T cells, Gr-1^+^CD11b^+^ granulocytes, CCR3^+^ eosinophils, CD4^+^CD25^+^ activated T cells, and B220^+^CD23^+^ B cells in a mouse model of asthma compared to control group, and the deficits in CCR3^+^ eosinophils were accompanied by concurrent decreases in eosinophils in BALF cytospin (Figures 4(d) and 4(e)). MA (300 mg/kg), MA128 (300 mg/kg), and CsA treatment with OVA resulted in significant reductions in CD4^+^ helper T cells (***P < 0.001), CD8^+^ c/s T cells (***P < 0.001), Gr-1^+^CD11b^+^ granulocytes (***P < 0.001), CD4^+^CD25^+^-activated T cells (**P < 0.01, ***P < 0.001), B220^+^CD23^+^ B cells (**P < 0.01, ***P < 0.001), CD4^+^ helper T cells (***P < 0.01, ***P < 0.001), Gr-1^+^CD11b^+^ granulocytes, CCR3^+^ eosinophils (***P < 0.01, ***P < 0.001), CD4^+^CD25^+^-activated T cells (*P < 0.05, **P < 0.01), and B220^+^CD23^+^ B cells (***P < 0.001) in BALF were also decreased significantly (Table 2).

Absolute numbers of various immune cell subtypes in lung and BALF were counted (described in Section 2). Results are expressed as mean ± S.E. (*N* = 8). Statistical significance between control and treatment groups was assessed by ANOVA (*P < 0.05, **P < 0.01, ***P < 0.001). N: normal Balb/c mice, CT (control): Ovalbumin inhalation + vehicle, OVA + CsA (10 mg/kg), OVA + MA (300 mg/kg), OVA + MA128 (300 mg/kg).

### 3.6. Inhibition of Th2 Cytokines (In Vivo and Ex Vivo), Eotaxin, and OVA-Specific IgE Production in BAL Fluid and Serum

**Figure 5:**
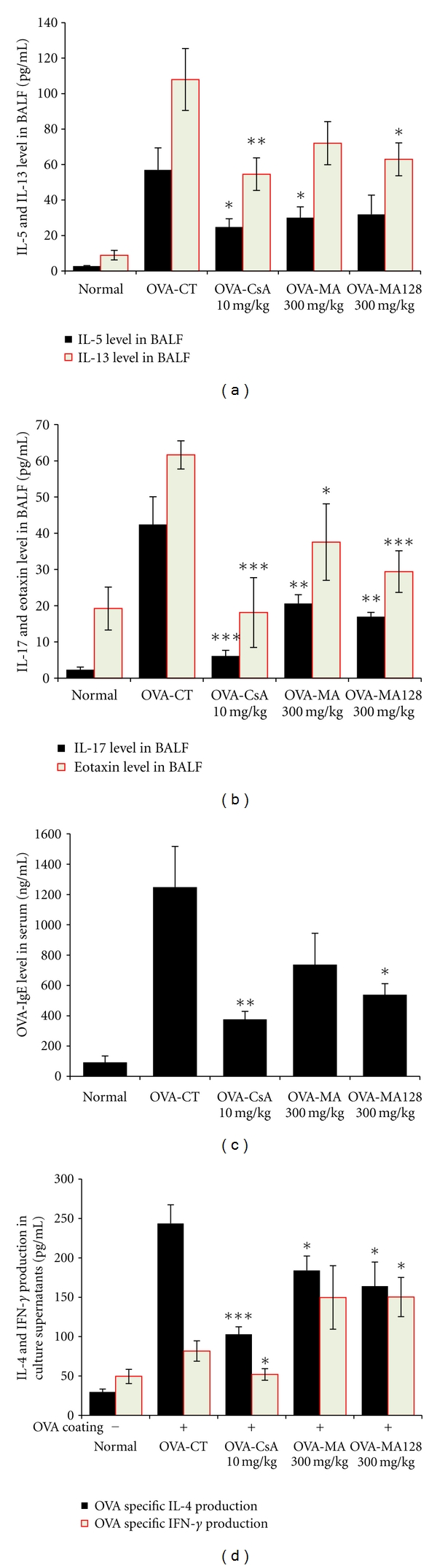
Cytokines, eotaxin, and ovalbumin-immunoglobulin E production in bronchoalveolar lavage fluid, OVA-specific splenocytes culture supernatant, and serum. (a), (b), and (c) Effect of MA and MA128 on Th2 cytokines (IL-5, IL-13), Th1 cytokine (IL-17), eotaxin in BALF and OVA-specific IgE in serum; (d) immunomodulatory effects of MA and MA128 on OVA-specific Th1/Th2 cytokines production in spleen cells (described in Section 2). Results are expressed the mean ± S.E. (*N* = 8). Statistical significance between control and treatment groups was assessed by ANOVA (*P < 0.05, **P < 0.01, significant increase; *P < 0.05). Normal: normal Balb/c mice, OVA-CT: Ovalbumin inhalation + vehicle, OVA + CsA (10 mg/kg), OVA + MA (300 mg/kg), OVA + MA128 (300 mg/kg).

The effect of MA and MA128 and CsA on Th2 cytokines and eotaxin protein levels was examined in BALF. As shown in Figures 5(a) and 5(b), IL-5, IL-13, IL-17, and eotaxin levels were significantly reduced in MA128-treated (300 mg/kg) and CsA-treated (10 mg/kg) mice. Also, IL-5, IL-17, and eotaxin levels were significantly reduced in MA-treated (300 mg/kg) mice. An important component of the allergic asthma model is the production of OVA-specific IgE. Therefore, levels of anti-OVA IgE were measured in serum from the OVA-challenged, PBS-, MA-, MA128-, and CsA-treated groups. In our study, OVA-specific IgE levels in serum from OVA-induced asthmatic mice were significantly increased compared with those from normal mice (PBS only), and MA128-treated (300 mg/kg) and CsA-treated (10 mg/kg) mice had significantly reduced OVA-specific IgE (Figure 5(c)). We also measured IL-4 and IFN-*γ* in the culture supernatants by ELISA and found that MA128 (300 mg/kg) and CsA (10 mg/kg) treatments significantly inhibited Th2 cytokine (IL-4) production in splenocytes (Figure 5(d)), which was accompanied by a concurrent decrease in Th2 cytokine production in BALF (Figure 5(a)).

## 4. Discussion

Several studies have demonstrated that certain traditional herbal medicine formulas have therapeutic benefits in allergic asthma [16]. In this murine model for chronic asthma [17], the changes in airway remodeling were characterized by epithelial hypertrophy, subepithelial fibrosis, and goblet cell hyperplasia that developed 4 to 6 weeks after allergen exposure. Many types of inflammatory cells, such as mast cells, eosinophils, and T lymphocytes [18], are involved in the process of airway inflammation. In addition, various cytokines and growth factors produced by these cells, such as interleukin (IL)-4, IL-5, IL-10, IL-12, and IL-13, may play important roles in the disease process [19].

We investigated whether MA and MA128 reduce the expression of remodeling markers such as goblet cell hyperplasia as well as of profibrogenic mediators in the lung such as eotaxin, IL-5, and IL-13. IL-5 and eosinophils have recently been specifically implicated as key contributors in the development of allergic airway inflammation. The predominance of Th2 phenotype of lymphocyte over Th1 phenotype from a very early stage of life has recently been highlighted, and this polarization likely has some relationship to the future development of allergic diseases such as asthma [20].

In our study, MA and MA128 reduced the productions of Th2 cell-associated cytokines, IL-4 and IL-5, and restored production of the Th1 cell-associated cytokine, IFN-*γ*, from CD4^+^ T cells prepared from lungs of sensitized mice compared to that in nonsensitized control mice. MA and MA128 also reduced the IL-4 levels and restored the IFN-*γ* levels in cultured supernatants from the spleen cells of sensitized mice. The changes in these cytokine levels in BAL fluid were no more dramatic than their productions from CD4^+^ T cells. It may be that the cytokines detected in BAL fluid originated from not only lung CD4^+^ T cells but also from other cells or serum.

Anti-OVA IgE antibody levels were reduced in the BAL fluids of the sensitized mice after oral administration of MA and MA128. It has been reported that Th2 cells encourage production of IgE antibody [21, 22]. These results indicate that MA and MA128 have a function that modulates the proliferation of lung Th cells from Th2-cell-dominant to Th1-cell-dominant in our murine airway inflammation model.

In our study, MA and MA128 did not reduce the anti-OVA IgE antibody level in the serum of these sensitized mice. Therapeutic agents that may be used in the treatment of asthma are numerous. Anti-IL-5 inhibits eosinophil adhesion, infiltration, and mediator release [23]. Eosinophilia is driven by allergen-activated Th2 cells that generate large amounts of Th2 cytokines (such as IL-4, IL-5, and IL-13). IL-5 is the most critical cytokine mediating increased eosinophil differentiation, activation, and survival [24]. The recruitment and activation of eosinophils appear to be controlled by the release of cytokines such as IL-5 and chemotactic agents such as eotaxin from Ag-stimulated T lymphocytes [25]. It has been suggested that IL-5 and eotaxin may collaborate in the regulation of blood and tissue eosinophilia in mice.

The mixture of medicinal herbs known as MA, which consists of *Sophora flavescens Aition*, *Arctii Fructus*, *Polygoni Cuspidati Radix*, *Lonicera japonica Thunberg*, and so forth, and its fermented product, MA128, has been widely used clinically for the treatment of allergic diseases including bronchial asthma and allergic rhinitis. The mechanism of MA and MA128 action in allergic diseases has been studied *in vitro* and *in vivo*. However, its mode of action has not been fully elucidated for bronchial asthma. 

In our preliminary study, MA and MA128 treatment (at 300 mg/kg) did not cause toxic effects on alanine aminotransferase and aspartic acid transaminase levels (data not shown). Therefore, we used that dose to study their effects in this model. Cyclosporin A can profoundly influence lymphocyte activation; it is thus appropriate to consider this drug as a novel antiasthma therapy [26]. In our study, we used CsA as a positive control for immunosuppressants. Recruitment of eosinophils to the airways is a characteristic of asthma, and the degree of eosinophilia is correlated with the severity of the disease. These cells are often considered to play a major role in inducing airway hyperresponsiveness (AHR) [27]. Eosinophilic inflammation is regulated to a major extent by activated T lymphocytes in the airways that secrete the Th2 cytokine IL-5 [28]. This cytokine is an important mediator in the regulation of eosinophilic inflammation through its effects on the proliferation, differentiation, and activation of eosinophils and as a signal for the rapid mobilization of eosinophils from the bone marrow [29].

In our study, MA and MA128 prevented the development of AHR, airway eosinophilia, and lung inflammation (Figures 2 and 3), and decreased Th2 cytokine levels in BAL fluid (Figure 5). These results demonstrate that MA and MA128 have profound regulatory effects on the development of lung allergic responses in the OVA-induced asthma model. Moreover, the regulatory effects exhibited by MA and MA128 were accompanied by the production of IL-4 in an *in vitro *assay (Figure 5(d)). Asthma produces immune abnormalities in a wide variety of cell populations. Thus, another goal in asthma research includes the evaluation of specific cell subpopulations. Immunophenotyping by flow cytometry showed a similar pattern as total lymphocyte numbers in BALF and lung. 

As previously described in the results, the effects of MA and MA128 on leukocyte subsets in lungs and BALF included marked changes in the numbers of CD4^+^ helper T cells, Gr-1^+^CD11b^+^ myeloid suppressor cells, CD3^−^CCR3^+^ eosinophils, CD4^+^CD25^+^-activated T cells, and B220^+^CD23^+^-activated B cells in a mouse model of asthma compared to control group (Table 2), and the deficits in CD3^−^CCR3^+^ eosinophils were accompanied by concurrent decreases in eosinophils in BALF cytospin (Figure 4(d)). MA and MA128 also inhibited B cell-dependent production of OVA-specific IgE in serum (Figure 5(c)), which correlated with the result of B220^+^CD23^+^ activated B cells in lung and BALF.

Such CC chemokines as eotaxin and RANTES induce preferential eosinophil recruitment in allergic inflammation. They also elicit proinflammatory effector functions of eosinophils, such as enhanced adhesion and superoxide generation. Chemokine-induced eosinophil degranulation, a major effector of eosinophil functions, is mediated through only CCR3, although some non-CCR3 ligands induce calcium influx in eosinophils. CCR3 may be an important target in the treatment of eosinophilic inflammation [30]. Eosinophils are attracted via their CCR3 to chemoattractants such as eotaxin released in the airways of asthmatics [31]. Inhibition of pulmonary eosinophilia by blocking the CCR3 receptor with antagonists may lead to a reduction in the inflammation and the airway responsiveness in asthma. This approach is being investigated by numerous research groups.

CD11b^+^Gr-1^+^, myeloid suppressor cells are referred to as myeloid-derived suppressor cells and are thought to be a heterogeneous cellular population containing macrophages, granulocytes, immature dendritic cells, and early myeloid precursors. Myeloid suppressor cells constitute a population of immature myeloid cells with potent immunosuppressive functions. They can induce immunosuppression under normal, inflammatory, or surgical/traumatic stress conditions [32]. The increase of Gr-1^+^CD11b^+^ myeloid-derived cells in allergic inflammation is related to OVA-induced asthma in mice [33].

In our study, CD11b^+^Gr-1^+^ myeloid suppressor cells were increased with OVA challenge but significantly decreased with MA and MA128 treatment (Table 1). Eotaxin was initially discovered using a biological assay designed to identify the molecules responsible for allergen-induced eosinophil accumulation in the lungs of guinea pigs [34]. The specific activity of all eotaxins is mediated by the selective expression of the eotaxin receptor, CCR3, a seven-transmembrane spanning, G protein-coupled receptor that is primarily expressed on eosinophils [35], and also noted on a subset of Th2 cells [36] and mast cells in humans [37]: CCR3 has been previously implicated in allergen-induced AHR [38]. However, the information regarding eotaxin and AHR is complex. In many cases, eotaxin caused substantial airway eosinophilia and, in conjunction with IL-5, and IL-13, caused an even more marked increase in eosinophils [39]. 

We observed significant correlations between eotaxin, IL-5, and IL-13 levels, and CCR3 expression on eosinophils. We hypothesize that MA and MA128 and CsA prevent AHR by downregulating eotaxin, IL-5 and IL-13 expression and, in so doing, by reducing eosinophilia. 

A recent study showed that the excessive production of interleukin (IL)-4, IL-5, and IL-13 by T-helper type 2 (Th2) cells is implicated in the development of asthma [40]. IL-5 mobilizes eosinophils from the bone marrow pool and chemokines such as eotaxin-1 induce the recruitment of eosinophils into the airway of experimental asthma models [41]. However, the T cell diversity has been expanded to several subpopulations, including T helper 17 (Th17) cells, suggesting that the mechanism is more complicated [42]. Interestingly, IL-17 is upregulated in asthma as eosinophils are cellular sources of its production, and IL-17 increases synthesis of IL-6 and IL-11 by bronchial fibroblasts derived from bronchial biopsies of asthmatic subjects. Several reports have demonstrated that TH_2_-type cytokines, including IL-4 and IL-13, enhance IL-17–induced release of IL-6 from fibroblasts, suggesting that IL-17 might be involved in asthma, which is a TH_2_-mediated disease [43]. Our results of increased expression of IL-17 in asthmatic samples support the role of IL-17 in asthma but do not explain its mechanism of action. To our knowledge, this expression of IL-17 in asthmatic lungs demonstrates the capacity of eosinophils to produce this potentially profibrotic cytokine. Therefore, our result can partly explain the reported inhibition of the Th2 cytokine (IL-5, IL-13), eotaxin, and IL-17 protein levels. A possible explanation could be that, in each specific mechanism, one or the other population can predominate and characterize the final result.

Therefore, MA and its fermented product, MA128, may reduce Th2 cytokine (IL-5, IL-13) production and gene expression by inhibiting IL-4 expression. Our result is not sufficient to explain the precise mechanism, which is more complicated. However, one possible mechanism is that MA and MA128 shifts immunity from a Th2 to a Th1 bias in the murine model of asthma. It would be interesting to precisely identify the complicated mechanisms of our results concerning Th1/Th2 transcription factors in future studies.

Recently, several studies have reported that certain traditional herbal medicine formulas for asthma have therapeutic benefits in allergic murine asthma model [44, 45]. Chung-Sang-Bo-Ha-Tang (CSBHT) [44] and Sho-seiryu-to (SST, Xiao-Qing-Long-Tang in Chinese) have been used to treat chronic asthma in Korea, China, and Japan for centuries [45]. The previous studies demonstrated that the approved effective dosages of CSBHT and SST for asthma were 1,000 mg/kg/day and 500 mg/kg/day while the dosages of MA and MA128 were 300 mg/kg/day. The comparison of the relative efficacy among these traditional herbal medicines was complicated due to the different sensitivities following the different experimental conditions, but the efficacies of MA and MA128 were likely to be stronger than those of CSBHT and SST. This result implies the possibility of using MA and MA128 for asthma medications.

## 5. Conclusion

The results of this study indicate that the herbal complex MA and its fermented product MA128 have antiasthmatic effects by suppressing eosinophil infiltration into airways and blood, allergic airway inflammation, and AHR, which occurred by suppressing the production of IL-5, IL-13, IL-17, Eotaxin, and OVA-specific IgE; by upregulating the production of OVA-specific Th1 cytokine (IFN-*γ*); and by downregulating OVA-specific Th2 cytokine (IL-4) in the culture supernatant of spleen cells. The effectiveness of MA was increased by fermentation with *Lactobacillus acidophilus*, which suggests that the biological activity of other herbal medicines might also be increased by fermentation with probiotics. Further study will elucidate the active bioconversion constituents and optimize the herbal composition and the fermentation process to increase the effectiveness.

## Figures and Tables

**Table 1:** Mobile phase condition of chromatographic separation.

Time (min)	Water	Acetonitrile	Flow rate (mL/min)
0	95	5	1
5	95	5	1
70	0	100	1
80	0	100	1

**Table 2:** Quantification by FACS analysis of various immune cell subtypes in lung and BALF.

	Cell phenotypes in lung & BAL	Normal	OVA-induced asthma mice (total absolute no.)			
		Balb/c mice	Control	CsA (10 mg/kg)	MA (300 mg/kg)	MA128 (300 mg/kg)
Lung	CD4^+^ (× 10^6^ cells)	1.0 ± 0.12	7.0 ± 0.17	2.3 ± 0.34***	4.0 ± 0.31***	3.4 ± 0.61***
	CD8^+^ (× 10^6^ cells)	1.1 ± 0.18	6.7 ± 0.60	1.2 ± 0.07***	3.8 ± 0.18***	2.8 ± 0.42***
	CD4^+^ CD25^+^ (× 10^6^ cells)	1.3 ± 0.15	11.5 ± 0.12	3.6 ± 0.06***	7.1 ± 1.09**	5.5 ± 0.61***
	B220^+^ CD23^+^ (× 10^6^ cells)	0.4 ± 0.09	4.9 ± 0.59	2.0 ± 0.26***	3.1 ± 0.01**	2.4 ± 0.25**
	CD11b^+^ Gr-1^+^ (× 10^6^ cells)	0.5 ± 0.05	7.7 ± 0.31	1.9 ± 0.17***	4.0 ± 0.20***	3.1 ± 0.38***

BAL	CD4^+^ (× 10^4^ cells)	0.8 ± 0.01	13.5 ± 1.03	4.6 ± 0.50***	9.6 ± 0.15**	6.8 ± 1.31***
	CCR3^+^ (× 10^4^ cells)	0.1 ± 0.05	4.2 ± 0.37	1.1 ± 0.07***	2.6 ± 0.01***	1.9 ± 0.47**
	CD4^+^ CD25^+^ (× 10^4^ cells)	0.4 ± 0.06	7.0 ± 1.23	2.6 ± 0.46**	4.4 ± 0.14*	3.9 ± 0.91*
	B220^+^ CD23^+^ (× 10^4^ cells)	0.1 ± 0.02	4.6 ± 0.17	1.3 ± 0.05***	202 ± 0.22***	1.7 ± 0.01***
